# Prolonged Length of Stay After One- and Two-Level Transforaminal Lumbar Interbody Fusion (TLIF): Risk Factors and Consequent Outcomes

**DOI:** 10.7759/cureus.91184

**Published:** 2025-08-28

**Authors:** Mitsuhiro Nishizawa, Steven D Glassman, Mladen Djurasovic, Charles H Crawford, Jeffrey Gum, John R Dimar, Kirk K Owens, Leah Carreon

**Affiliations:** 1 Orthopedic Surgery, University of Louisville School of Medicine, Louisville, USA; 2 Orthopedics, Norton Healthcare, Norton Leatherman Spine Center, Louisville, USA; 3 Research, Norton Healthcare, Norton Leatherman Spine Center, Louisville, USA

**Keywords:** complications, length of stay, oswestry disability index, patient-reported outcomes, tlif, transforaminal lumbar interbody fusion

## Abstract

Summary of background data: While many studies have assessed factors associated with prolonged length of stay (LOS) following spine surgery, a limited number of studies have large cohorts with granular individual patient-level data, and little is known about the association between prolonged LOS and functional recovery after spine surgery.

Objective: We aim to identify factors associated with prolonged length of stay (LOS) and its impact on postoperative complications and functional recovery.

Methods: The present study is a retrospective longitudinal observational cohort study. Patients (N=576) who underwent one- or two-level transforaminal lumbar interbody fusion (TLIF) were identified. LOS was measured in hours, and prolonged LOS was defined as greater than the 75th percentile (>85 hours). Demographic and surgical parameters, and in-hospital and post-discharge complications were collected. Patient-reported outcome measures (PROMs), including the Oswestry Disability Index (ODI) and Numeric Rating Scale (NRS) scores for back and leg pain, were collected preoperatively and at six weeks, three months, and one year postoperatively. Multiple logistic regression identified factors associated with prolonged LOS and post-discharge complications.

Results: The mean LOS for the entire cohort was 75.0±50.1 hours, with 145 patients categorized as prolonged LOS (141.4±54.5 hours). Prolonged LOS was independently associated with older age (odds ratio (OR)=1.03, p=0.003), lower preoperative hemoglobin (OR=0.80, p=0.020), longer operative time (OR=1.01, p<0.001), and in-hospital complications (OR=11.09, p<0.001). Prolonged LOS was also associated with higher post-discharge complication rates, including 30-day readmission (14% versus 6%, p=0.004), surgical site infection (6% versus 1%, p=0.002), and deep vein thrombosis (DVT)/pulmonary embolism (PE) (3% versus 0%, p=0.001). As complications are known to prolong LOS, a sub-analysis of 205 patients with complete PROMs and no post-discharge complications was performed. In this sub-analysis, patients with prolonged LOS had significantly worse preoperative ODI (51.8 versus 48.5, p=0.025), back pain (7.1 versus 6.2, p=0.013), and leg pain (6.8 versus 5.9, p=0.035). Postoperatively, improvement in PROMs was worse in prolonged LOS patients at all time points.

Conclusion: Prolonged LOS after one- or two-level TLIF was associated with older age, lower preoperative hemoglobin, longer operative time, and in-hospital complications. It was also associated with a higher risk of post-discharge complications and lower functional status.

## Introduction

Transforaminal lumbar interbody fusion (TLIF) is a common procedure for treating degenerative low back conditions [1]. With the aging population, the need for lumbar fusion surgery has risen [2,3]. The cases have increased by 62.3% from 2004 to 2015 and accounted for nearly 40% of all spine surgery fusion costs, totaling $10.2 billion in 2015. The hospital cost is linked to hospitalization costs, with the cost per admission increasing by 70%, reaching $51,601 from 2004 to 2015 [4].

Hospital length of stay (LOS) is linked to hospital cost and is a key indicator of the quality and efficiency of patient care [5-7]. Prolonged LOS negatively impacts both healthcare costs and patient health. It is reported that prolonged LOS increases the risk of adverse events such as hospital-acquired infections, deep vein thrombosis (DVT), surgical site infection, unplanned readmissions, and even mortality [8-10]. Many studies have identified risk factors for prolonged LOS after lumbar fusion surgery, including advanced age, dependent functional status, and longer operative time [11-14]. Despite this, associations between prolonged hospitalization and delayed functional recovery after lumbar fusion surgery have not been previously studied. Delayed functional recovery has implications for the clinician and the patient, as return to function is one of the aims of elective lumbar surgery for degenerative disorders.

Many studies utilized national or large administrative databases, resulting in a lack of granularity and enough information to evaluate risk factors [11-16]. A limited number of studies have comprehensively evaluated prolonged LOS from preoperative predictors to post-surgical sequelae using granular data, but none have evaluated functional recovery, as individual-level functional outcome measures are not available in these administrative databases. Moreover, no studies have assessed the association between prolonged LOS and functional recovery following lumbar fusion surgery. Thus, this study aims to fill these gaps by assessing the factors contributing to prolonged LOS and its impact on surgical outcomes and functional recovery. Specifically, we aim to identify independent risk factors for prolonged LOS following lumbar fusion surgery, to analyze the association between prolonged LOS and post-discharge complications, and to evaluate the impact of prolonged LOS on patient-reported functional outcomes even in the absence of complications.

## Materials and methods

We collected data from clinical records of a consecutive series of patients who underwent one- or two-level open TLIF for lumbar disc herniation, spondylosis, or degenerative spondylolisthesis at a single institution between January 2013 and December 2021. Patients who had emergent or urgent surgery, surgery for infection, tumor, or trauma were excluded. TLIF cases were selected as the study population, as this is an elective procedure with a low incidence of complications [1]. Patients who had an anterior, lateral, oblique, or extreme lateral lumbar interbody fusion or had a posterior or posterolateral only fusion were excluded.

Standard demographics, including age, sex, body mass index (BMI), smoking status, diabetes mellitus, American Society of Anesthesiologists (ASA) grade, and history of prior lumbar spine surgery, and surgical parameters, including the levels of interbody fusion, operative time, and estimated blood loss (EBL), were collected [17]. LOS was measured in hours, and prolonged LOS was defined as greater than the 75th percentile (>85 hours) based on definitions used in previous studies [13,18].

Perioperative complications, including surgical complications (e.g., dural tear and EBL > 1,000 mL) and in-hospital complications, and post-discharge complications within one year after surgery, were recorded by an individual not involved in the patient’s care from the electronic medical record. Patient-reported outcome measures (PROMs), including the Oswestry Disability Index (ODI) and Numeric Rating Scale (NRS) scores for back and leg pain, were collected preoperatively and at six weeks, three months, and one year postoperatively [19-21] during the patient’s routine clinical visit.

Statistical analysis

Continuous variables were summarized as the mean±standard deviation (SD), and categorical variables as frequencies with percentages. The Shapiro-Wilk test was used to assess the normality of continuous variables. Missing data was not imputed.

The demographics, surgical parameters, and complication rates were compared between patients with prolonged LOS (prolonged LOS) and those without (standard LOS). Student’s t-test was used to compare groups for normally distributed continuous variables, and the Mann-Whitney U test was applied for non-normally distributed continuous variables. Categorical variables were compared using Fisher’s exact test. Multiple logistic regression was used to identify the factors associated with prolonged LOS. In the model, we included risk factors that were deemed of clinical importance to contribute to prolonged LOS or had a univariate association of p<0.10. As a subgroup analysis, PROMs were analyzed in patients without perioperative or postoperative complications, accounting for the known impact of complications on PROMs [22]. These were then compared between groups at six weeks, three months, and one year. Statistical significance was defined as p<0.05. All analyses were performed using R software (version 4.4.2).

This study was reviewed and approved by the University of Louisville Institutional Review Board, which determined that the study is exempt according to 45 CFR 46.101(b) under Category 4: Secondary research for which consent is not required: Secondary research uses of identifiable private information or identifiable biospecimens.

## Results

A total of 576 patients were included in this study. The mean age was 58.7±12.0 years, and there were 335 (61%) female patients. The mean LOS for the entire cohort was 75.0±50.1 hours, and a total of 145 patients were categorized as prolonged LOS (mean: 141.4±54.5 hours).

In univariate analysis, patients with prolonged LOS were significantly older (62.5±11.4 versus 57.4±11.9, p<0.001), had a higher incidence of diabetes (29% versus 19%, p=0.014), had previous lumbar surgery (32% versus 23%, p=0.046), and had higher ASA grade (2.8±0.5 versus 2.6±0.5, p<0.001). They also had lower preoperative hemoglobin (13.3±1.4 versus 14.0±1.4, p<0.001), higher EBL (384.9±280.4 versus 271.1±207.0, p<0.001), longer operative time (219.5±67.7 versus 187.9±55.6, p<0.001), and a higher incidence of overall perioperative complications (37% versus 8%, p<0.001), intraoperative complications (12% versus 4%, p=0.004), and in-hospital complications (28% versus 4%, p<0.001), compared to patients with standard LOS (Table 1).

**Table 1:** Comparison of patient demographics and surgical data between patients with prolonged length of stay (prolonged LOS) and those without (standard LOS) LOS: length of stay, SD: standard deviation, BMI: body mass index, ASA: American Society of Anesthesiologists, EBL: estimated blood loss

Demographics and surgical data	Prolonged LOS	Standard LOS	p-value
Number of patients, number (%)	145 (25%)	431 (75%)	-
Age, years, mean (SD)	62.5 (11.4)	57.4 (11.9)	<0.001
Female, number (%)	98 (68%)	255 (59%)	0.077
BMI, kg/m^2^, mean (SD)	33.1 (7.1)	32.0 (6.8)	0.147
Current smoker, number (%)	23 (16%)	84 (19%)	0.396
Diabetic, number (%)	42 (29%)	82 (19%)	0.014
ASA grade, mean (SD)	2.8 (0.5)	2.6 (0.5)	0.001
Surgical history, number (%)	46 (32%)	99 (23%)	0.046
Number of fusion level, number (%)			>0.999
One	128 (88%)	380 (88%)	
Two	17 (12%)	51 (12%)	
Preoperative hemoglobin, g/dL, mean (SD)	13.3 (1.4)	14.0 (1.4)	<0.001
EBL, mL, mean (SD)	384.9 (280.4)	271.1 (207.0)	<0.001
Operative time, minutes, mean (SD)	219.5 (67.7)	187.9 (55.6)	<0.001
LOS, hours, mean (SD)	141.4 (54.5)	52.6 (19.3)	<0.001
Overall perioperative complications, number (%)	53 (37%)	36 (8%)	<0.001
Intraoperative complications, number (%)	18 (12%)	21 (5%)	0.004
Dural tear, number (%)	15 (10%)	17 (4%)	0.006
EBL > 1,000 mL, number (%)	3 (2%)	4 (0.9%)	0.376
In-hospital complications, number (%)	40 (28%)	15 (4%)	<0.001

Multiple regression analysis showed that prolonged LOS was independently associated with age (odds ratio (OR)=1.03, 95% confidence interval (CI)=1.01-1.06, p=0.003), preoperative hemoglobin (OR=0.80, 95% CI=0.66-0.96, p=0.020), operative time (OR=1.01, 95% CI=1.00-1.01, p<0.001), and in-hospital complications (OR=11.09, 95% CI=5.64-22.97, p<0.001) (Table 2).

**Table 2:** Multiple logistic regression analysis: prolonged length of stay OR: odds ratio, CI: confidence interval, BMI: body mass index, ASA: American Society of Anesthesiologists

Parameter	OR (95% CI)	p-value
Age, years	1.03 (1.01-1.06)	0.003
Female	1.31 (0.75-2.23)	0.324
BMI, kg/m^2^	1.01 (0.98-1.04)	0.547
ASA	1.25 (0.80-2.06)	0.358
Diabetic	1.61 (0.94-2.67)	0.073
Surgical history	1.62 (0.65-1.77)	0.244
Preoperative hemoglobin, g/dL	0.80 (0.66-0.96)	0.020
Estimated blood loss, mL	1.00 (0.99-1.00)	0.103
Operative time, minutes	1.01 (1.00-1.01)	<0.001
Intraoperative complications	1.62 (0.70-3.63)	0.243
In-hospital complications	11.09 (5.64-22.97)	<0.001

Among in-hospital complications, patients with cardiovascular events and deep vein thrombosis (DVT)/pulmonary embolism (PE) or those requiring return to the operating room (OR) had the longest LOS, with a mean of 162.3±138.0 hours, 163.5±16.3 hours, and 162.2±45.0 hours, respectively (Table 3).

**Table 3:** Perioperative complications and mean LOS LOS: length of stay, SD: standard deviation, EBL: estimated blood loss, OR: operating room, DVT/PE: deep vein thrombosis/pulmonary embolism, GI: gastrointestinal

Parameter	Frequency (%)	LOS, hours, mean (SD)
Overall perioperative complications	89 (15%)	116.7 (72.0)
Intraoperative complications	39 (7%)	100.6 (54.4)
Dural tear	32 (6%)	103.2 (55.1)
EBL > 1,000 mL	7 (1%)	88.9 (53.7)
In-hospital complications	55 (10%)	131.9 (78.8)
Return to OR	8 (1%)	162.2 (45.0)
Implant malposition	3 (0.5%)	183.0 (51.9)
Evacuation of hematoma/seroma	5 (0.9%)	149.8 (41.1)
Cardiovascular	10 (2%)	162.3 (138.0)
Pulmonary	3 (0.5%)	149.3 (123.8)
DVT/PE	2 (0.3%)	163.5 (16.3)
Electrolyte abnormalities	7 (1%)	137.6 (40.3)
Urinary retention	15 (3%)	135.7 (117.0)
GI complications	5 (0.9%)	135.4 (78.9)
Anemia requiring transfusion	3 (0.5%)	127.7 (72.0)
Neurologic/altered mental status	6 (1%)	118.8 (19.7)
Urinary tract infection	4 (0.7%)	113.0 (69.7)
Other	3 (0.5%)	65.7 (58.3)

A total of 120 (21%) patients experienced post-discharge complications within one year postoperatively. Prolonged LOS was associated with significantly higher rates of 30-day readmission (14% versus 6%, p=0.004), 90-day readmission (9% versus 4%, p=0.034), surgical site infection (6% versus 1%, p=0.002), cardiovascular complications (6% versus 1%, p=0.005), DVT/PE (3% versus 0%, p=0.001), and neurologic complications (6% versus 0.5%, p<0.001), compared to those with standard LOS (Table 4).

**Table 4:** Comparison of complication rate within one year after discharge between patients with prolonged LOS (prolonged LOS) and those without (standard LOS) LOS: length of stay, DVT/PE: deep vein thrombosis/pulmonary embolism, GI: gastrointestinal

Parameter	Prolonged LOS	Standard LOS	p-value
Number of patients, number (%)	145 (25%)	431 (75%)	-
Overall complications, number (%)	34 (23%)	86 (20%)	0.409
30-day readmission	20 (14%)	25 (6%)	0.004
90-day readmission	13 (9%)	18 (4%)	0.034
Surgical site infection	9 (6%)	5 (1%)	0.002
Wound dehiscence	2 (1%)	6 (1%)	>0.999
Cardiovascular	8 (6%)	5 (1%)	0.005
Pulmonary	5 (3%)	2 (0.5%)	0.013
DVT/PE	5 (3%)	0 (0%)	0.001
GI complications	6 (4%)	8 (2%)	0.128
Neurologic/altered mental status	8 (6%)	2 (0.5%)	<0.001
Urinary tract infection	2 (1%)	3 (0.7%)	0.604
Other	4 (3%)	5 (1%)	0.239

In a subgroup analysis, PROMs were analyzed in 205 patients without complications and with completed PROMs up to one year postoperatively (36%). Although 371 (64%) patients were excluded from this analysis, a sensitivity analysis showed no significant differences in demographics, surgical parameters, or preoperative PROMs between included and excluded patients. Among the 205 patients, those with prolonged LOS had significantly worse preoperative ODI (51.8±18.1 versus 48.5±16.6, p=0.025), back pain (7.1±2.2 (prolonged LOS) versus 6.2±2.3 (standard LOS), p=0.013), and leg pain (6.8±2.6 (prolonged LOS) versus 5.9±2.6 (standard LOS), p=0.035), compared to those with standard LOS (Table 5).

**Table 5:** Comparison of PROMs, including the ODI and NRS pain scores (back pain and leg pain), measured preoperatively and at three months and one year postoperatively between patients with prolonged length of stay (prolonged LOS) and those without (standard LOS) in patients with PROMs data and no post-discharge complications PROMS: patient-reported outcome measures, ODI: Oswestry Disability Index, NRS: Numeric Rating Scale, LOS: length of stay

Parameter	Prolonged LOS	Standard LOS	p-value
Number of patients	52	153	-
ODI			
Preoperative	56.2 (17.3)	47.0 (15.4)	0.038
6 weeks	46.7 (20.3)	34.1 (19.4)	<0.001
3 months	39.8 (20.1)	28.4 (19.9)	<0.001
1 year	37.6 (20.2)	28.7 (20.2)	0.003
Back pain			
Preoperative	7.3 (2.0)	6.1 (2.3)	0.013
6 weeks	4.5 (2.9)	3.3 (2.6)	0.008
3 months	4.3 (2.7)	2.8 (2.6)	<0.001
1 year	4.3 (2.7)	3.4 (2.7)	0.003
Leg pain			
Preoperative	7.1 (2.4)	5.9 (2.3)	0.035
6 weeks	3.9 (3.2)	2.2 (2.6)	0.001
3 months	4.0 (3.2)	2.0 (2.5)	<0.001
1 year	3.9 (2.9)	2.7 (2.8)	<0.001
Changes from preoperative to postoperative			
Δ ODI			
6 weeks	-11.0 (24.5)	-15.5 (19.8)	0.023
3 months	-18.1 (26.7)	-19.9 (19.9)	0.034
1 year	-21.7 (26.9)	-19.8 (19.8)	0.062
Δ Back pain			
6 weeks	-3.3 (3.0)	-3.1 (3.0)	0.242
3 months	-3.3 (2.8)	-3.6 (3.1)	0.049
1 year	-3.4 (3.2)	-3.0 (3.1)	0.305
Δ Leg pain			
6 weeks	-3.7 (3.3)	-3.1 (3.0)	0.078
3 months	-3.2 (3.9)	-4.2 (3.2)	0.014
1 year	-3.4 (3.2)	-3.5 (3.4)	0.213

Postoperatively, significant differences remained for all of them at any time point. The magnitude of changes in scores from preoperative to postoperative significantly differed in ODI at six week (-9.3±22.1 (prolonged LOS) versus -15.6±19.3 (standard LOS) p=0.023) and three months (-14.5±24.2 (prolonged LOS) versus -20.2±19.7 (standard LOS), p=0.034), back pain at three months (-2.8±2.8 (prolonged LOS) versus -3.6±3.1 (standard LOS), p=0.049), and leg pain at three months (-2.9±3.9 (prolonged LOS) versus -4.3±3.2 (standard LOS), p=0.014).

## Discussion

This study offers a comprehensive analysis of prolonged LOS, from identifying risk factors to assessing subsequent outcomes, including post-discharge complications and functional recovery, providing a complete picture of prolonged LOS.

Our study identified older age, lower preoperative hemoglobin, longer operative time, and in-hospital complications as independent risk factors for prolonged LOS following lumbar fusion surgery. These findings are consistent with previous studies that evaluated factors associated with prolonged LOS after lumbar fusion surgery using multiple regression analysis and address the gap in the literature. Gruskay et al. reported that postoperative complications were an independent risk factor of prolonged LOS in a study of 103 patients; however, the small sample size presented a limitation [12]. In a multicenter study of 1,168 patients with lumbar fusion surgery, Kobayashi et al. identified older age, longer operative time, and higher EBL as risk factors for prolonged LOS [18]. However, the mean LOS in the study was 21 days, which was much longer than that in our study (less than three days), indicating potential differences in the populations or conditions of the patients. Additionally, preoperative anemia was identified as an independent risk factor for prolonged LOS in lumbar decompression surgery in the study by Phan et al. [23] using a national database, as well as in other orthopedic procedures [24].

The association between prolonged LOS and readmission rates after spine surgery has been well-studied in previous literature. Akamnonu et al. reported that prolonged LOS was associated with a higher 90-day readmission rate in their study of 1,306 patients after lumbar spine surgery [16]. Similarly, Kim et al. found, in a study of 33,840 propensity score-matched patients, that prolonged LOS independently increased the risk of unplanned 30-day readmissions [25]. Additionally, they also found that prolonged LOS was associated with an increased risk of surgical site infection, cardiovascular complications, pulmonary issues, stroke, and DVT/PE. In our study, prolonged LOS was also associated with 30-day and 90-day readmission rates, surgical site infections, cardiovascular and pulmonary complications, DVT/PE, and neurologic events, aligning with these previous findings.

Furthermore, our study is the first to demonstrate that prolonged LOS was a marker for worse postoperative PROMs, even in patients who did not experience complications after discharge. Although this study cannot determine a causal relationship, possible explanations include the impact of immobility due to prolonged LOS. Lack of physical activity can significantly impair not only cardiovascular, respiratory, immune, and cognitive systems but also musculoskeletal [26,27]. Muscle wasting occurs quickly with immobility, starting as early as 24 hours after bed rest in an inflammatory condition [28]. The erector muscles, including the spinal extensors and quadriceps, are particularly susceptible to wasting from prolonged bed rest and require a longer recovery time compared to the flexors [29,30].

The study has several limitations. First, it is a retrospective study conducted at a single institution, making it difficult to control for potential confounding factors, including patient socioeconomic status, case start time, different anesthesia regimens, lack of standardized rehabilitation protocols, postoperative pain management, or potential surgeon- and technique-related variability. Second, we defined prolonged LOS in the same manner as previous literature (>75th percentile), but LOS can vary greatly depending on the medical system, facility, or country, which may limit the generalizability of our findings [13,18]. Third, we did not record hospitalizations or emergency department visits at hospitals outside our system. Fourth, the relatively small numbers in subgroups (e.g., DVT/PE and neurologic complications) may limit the reliability of associations with specific complications. Fifth, not all patients had a full follow-up set of PROMs data available. Lastly, long-term outcomes beyond one year are absent, reducing insight into sustained recovery. Despite these limitations, our study is notable for providing a comprehensive picture of prolonged LOS, including its risk factors and consequences following lumbar fusion surgery. Despite these limitations, our study is remarkable in providing a comprehensive picture of prolonged LOS, including its risk factors and consequences following TLIF.

## Conclusions

Prolonged LOS after one- or two-level TLIF was associated with older age, lower preoperative hemoglobin, longer operative time, and in-hospital complications. It was also associated with a higher risk of post-discharge complications and lower functional status. However, prolonged LOS was associated not only with complications but also with diminished long-term functional outcomes even in the absence of complications. Patients with prolonged hospital stays, even without complications, should be counseled to expect the possibility of delayed functional recovery after TLIF surgery and may be candidates for aggressive early post-discharge rehabilitation. Further studies are necessary to explore the associations and causes for delayed functional recovery in patients with prolonged hospitalizations after TLIF.
